# Structural Changes of Bagasse during the Homogeneous Esterification with Maleic Anhydride in Ionic Liquid 1-Allyl-3-methylimidazolium Chloride

**DOI:** 10.3390/polym10040433

**Published:** 2018-04-13

**Authors:** Huihui Wang, Wei Chen, Xueqin Zhang, Yi Wei, Aiping Zhang, Shijie Liu, Xiaoying Wang, Chuanfu Liu

**Affiliations:** 1 State Key Laboratory of Pulp and Paper Engineering, South China University of Technology, Guangzhou 510640, China; wang.huihui@mail.scut.edu.cn (H.W.); geogeo_chen@163.com (W.C.); xueqin0228@gmail.com (X.Z); fewvergil@mail.scut.edu.cn (Y.W.); xyw@scut.edu.cn (X.W.); 2 College of Forestry and Landscape Architecture, South China Agricultural University, Guangzhou 510642, China; aiping@scau.edu.cn; 3 Department of Paper and Bioprocess Engineering, College of Environmental Science and Forestry, State University of New York, Syracuse, NY 13210, USA

**Keywords:** bagasse, maleic anhydride, structural changes, AmimCl

## Abstract

The maleation of bagasse could greatly increase the compatibility between bagasse and composite matrixes, and the percentage of substitution (PS) of bagasse maleates could be regulated in the homogeneous system. However, due to the complicated components and the linkages of bagasse, it was difficult to control the reaction behaviors of each component. In this paper, the detailed structural changes of bagasse during the homogeneous maleation in ionic liquid 1-allyl-3-methylimidazolium chloride (AmimCl) were comparatively investigated with the three main components (cellulose, hemicelluloses, and lignin) from bagasse. The PS of the maleated bagasse was 12.52%, and the PS of the maleated cellulose, hemicelluloses, and lignin were 13.50%, 10.89%, and 14.03%, respectively. Fourier translation infrared (FT-IR) and NMR analyses confirmed that the three main components were all involved in the homogeneous maleation. ^1^H-^13^C HSQC analysis indicated that the predominant monoesterification of cellulose, diesterification of hemicelluloses and lignin, and the degradation of the three main components simultaneously occurred. Besides, the quantitative analysis from ^1^H-^13^C HSQC revealed the relative PS of reactive sites in each component. ^31^P NMR results showed that the reactivity of lignin aliphatic hydroxyls was higher than that of phenolic ones, and the reactivity of phenolic hydroxyls followed the order of *p*-hydroxyphenyl hydroxyls > guaiacyl hydroxyls > syringyl hydroxyls.

## 1. Introduction

Biomass resources with low cost, renewability, and biodegradability, have attracted much attention to cope with concerns about the depletion of fossil resources and the environmental impact of petroleum-based plastics [1,2]. Bagasse, a byproduct from the sugar industry, is a kind of currently underutilized lignocellulosic biomass. The development of ways to convert bagasse into new value-added materials will greatly influence biorefinery economics [3,4]. The use of bagasse as inexpensive structural fillers in composite materials is one of the most economic application methods and has been widely explored. For example, bagasse ash was used as reinforcing fillers in thermoplastic materials, which had similar properties of commercial silica [5]. Polypropylene matrix composites reinforced with bagasse fiber showed increased mechanical properties by hot compression [6]. Additionally, poly (vinyl alcohol) blended with bagasse seemed to improve the biodegradation propensity [7]. However, bagasse without any derivatization often negatively impacts the compatibility between bagasse and composite matrixes [8,9,10].

The poor compatibility between unmodified bagasse and composite matrixes was attributed to the complicated structures and complex linkages, among various components [11,12]. Chemical modification could introduce functional groups and allow for novel materials with tailored properties to be designed [13]. Esterification is one of the most common and attractive modification methods, especially maleation. It has been reported that maleated bagasse could improve the compatibility, mechanical properties, and diffusivity of composites [9,14,15]. The application of maleic anhydride as esterification reagent produced no byproducts [16,17]. Besides, maleic anhydride could be derived from furfural or 5-hydroxyfurfural, which could be widely derived from hemicelluloses or cellulose [18,19,20].

According to the previous literatures [21,22], homogeneous reaction systems could greatly facilitate the esterification efficiency. Because of the high thermal stability, negligible vapor pressure, low flammability, and recyclability, ionic liquids (ILs) have attracted much attention among various solvents of lignocellulose [23]. Additionally, ILs can be tailored to meet the required application due to its designability [24]. Recently, ILs as efficient solvents have been widely studied [25,26], and the derivatization of bagasse in ILs has been reported [27,28,29]. There also have been some literatures on the homogeneous esterification between bagasse and maleic anhydride in ILs [21,30]. However, due to its complicated components and complex linkages, the detailed structural changes of each component during the homogeneous maleation of bagasse, to our knowledge, have not been attempted.

In this paper, bagasse was esterified with maleic anhydride comparatively with the three main components (cellulose, hemicelluloses, and lignin) to reveal the detailed structural changes during the homogeneous maleation in 1-allyl-3-methylimidazolium chloride (AmimCl). Samples were characterized with Fourier translation infrared (FT-IR), one-dimensional (1D) (^1^H, ^13^C, and ^31^P), and two-dimensional (2D) (^1^H-^1^H correlation spectroscopy (COSY), ^1^H-^13^C heteronuclear single-quantum correlation (HSQC), heteronuclear multiple-bond correlation (HMBC)) nuclear magnetic resonance (NMR), and X-ray photoelectron spectroscopy (XPS).

## 2. Materials and Methods

**Materials.** Bagasse was provided by a local factory (Jiangmen, China). It was air-dried, ground, and screened to obtain 20–40 mesh size particles (450–900 µm). The ground bagasse was extracted with toluene-ethanol (2:1, *v*/*v*) for 4 h, and then air-dried in a cabinet oven at 50 °C for 24 h. According to our previous study [31], the contents of cellulose, hemicelluloses, and lignin in the extractive-free bagasse were 44.85%, 33.13%, and 19.24%, respectively.

AmimCl was purchased from the Shanghai Cheng Jie Chemical Co. Ltd. (Shanghai, China). Cellulase from *Trichoderma viride* (3–10 units/mg solid) was purchased from Sigma-Aldrich Co. (Shanghai, China). Maleic anhydride and other chemicals were analytical grade, and purchased from Guangzhou Chemical Reagent Factory (Guangzhou, China).

**The Isolation of Cellulose, Hemicelluloses, and Lignin from Bagasse.** Cellulose, hemicelluloses, and lignin were isolated from the extractive-free bagasse, according to our previous literature [31].

**Homogeneous Maleation in AmimCl.** The isolated cellulose and extractive-free bagasse (12 g) were finely ball-milled with two 500 mL ZrO_2_ jars with ZrO_2_ balls (total 801 g) for 4 h in a planetary BM4 ball-mill (Grinder, Beijing, China) at 220 rpm. Hemicelluloses and lignin were directly used without ball-milling. The materials (0.4 g) were dispersed in AmimCl (20 g) without heating under the protection of N_2_. The mixture was heated at 90 °C under magnetic agitation for 4 h to obtain a clear solution. Maleic anhydride (2.0 g) was added to the solution with the weight ratio of 1:5 (material/maleic anhydride, *g*/*g*). The reaction was carried out at 90 °C for 90 min with magnetic agitation under nitrogen atmosphere. After the required time, the flask was cooled to room temperature, and the products were precipitated and washed with ethanol (99.5 wt %, total 800 mL). The residues, after filtration, were freeze-dried for further characterization.

**Determination of the Percentage of Substitution (PS).** The theoretical hydroxyl contents of unmodified cellulose and hemicelluloses were calculated, according to the reported literature [32], and the substituted hydroxyl contents of the maleated bagasse, cellulose, and hemicelluloses were determined by the back-titration method [32].

The hydroxyl contents of lignin samples were determined with ^31^P NMR, according to the previous literature [33], and were calculated based on Equation (1).

(1) $$A=\frac{\frac{\rho \times 100\times 10^{-6}}{100.16}\times \frac{A_{2}}{A_{1}}}{m}$$

where *A* (mmol/g) is the hydroxyl content of lignin sample, *A*_2_ is the integration of resonance assigned to hydroxyls of lignin, *A*_1_ is integration of resonance assigned to hydroxyls of cyclohexanol, *ρ* (mg/mL) is the concentration of cyclohexanol, *m* (g) is the dry weight of lignin sample, 100 (μL) is the consumed volume of cyclohexanol, and 100.16 (g/mol) is the molar mass of cyclohexanol.

The theoretical hydroxyl content of unmodified bagasse was calculated according to the reported literature [32]. The *PS* of sample was calculated based on Equation (2).

(2) $$PS=\frac{n^{\prime }{}_{OH}}{n_{OH}}\times 100\%$$

where *n_OH_* (mmol/g) is the theoretical hydroxyl content of unmodified sample, *n’_OH_* (mmol/g) is the substituted hydroxyl content of maleated sample, and *PS* is the percentage of substitution.

**Analytical Methods.** FT-IR spectra were collected on Tensor 27 spectrometer (Bruker, Karlsruhe, Germany) in the range of 4000–400 cm^−1^ with 32 scans [34].

The ^1^H NMR, ^1^H-^1^H COSY, ^13^C NMR, ^1^H-^13^C HSQC, and ^1^H-^13^C HMBC NMR spectra were recorded on a Bruker Advance III 600 MHz spectrometer (Bruker, Karlsruhe, Germany) at 298.0 K from the dried samples (40–50 mg) dissolved in 0.5 mL of DMSO-*d*_6_. XPS was conducted on EscaLab 250Xi (ThermoFisher Scientific, Waltham, MA, USA) with a Al-Kα radiation source gun. The detailed instrumental parameters were listed in the supplementary material.

## 3. Results and Discussion

### 3.1. PS of the Maleated Bagasse

The theoretical hydroxyl content of unmodified bagasse was calculated as 14.30 mmol/g, based on the reported method [32]. After maleation, the substituted hydroxyl content of bagasse was 1.79 mmol/g, corresponding to the PS 12.52%. When compared with maleated bagasse that was prepared in 1-butyl-3-methylimidazolium chloride [30], the PS of the maleated bagasse in AmimCl in the present study was reasonable. However, it was hard to distinguish the reaction behaviors of each component during the homogeneous maleation due to the complicated structures, components and linkages. Therefore, the three main bagasse components (cellulose, hemicelluloses, and lignin) were comparatively maleated under the same conditions as bagasse. The theoretical hydroxyl contents of unmodified cellulose and hemicelluloses were 18.52 and 15.15 mmol/g, according to the reported method [32], and the theoretical hydroxyl content of unmodified lignin that was calculated from ^31^P NMR spectrum was 5.06 mmol/g. After maleation, the hydroxyl contents of the maleated cellulose, hemicelluloses, and lignin decreased to 16.02, 13.50, and 4.32 mmol/g, respectively. The substituted hydroxyl contents of the maleated cellulose, hemicelluloses, and lignin were 2.50, 1.65, and 0.71 mmol/g, respectively, corresponding to the PS 13.50%, 10.89%, and 14.03%, as shown in Table 1. These results indicated that the three main components were all involved in the homogenous maleation. Besides, it also indicated that the reactivity of bagasse components during the homogeneous maleation followed the order of lignin > cellulose > hemicelluloses [35].

### 3.2. Structural Changes of the Maleated Bagasse

**Figure 1:**
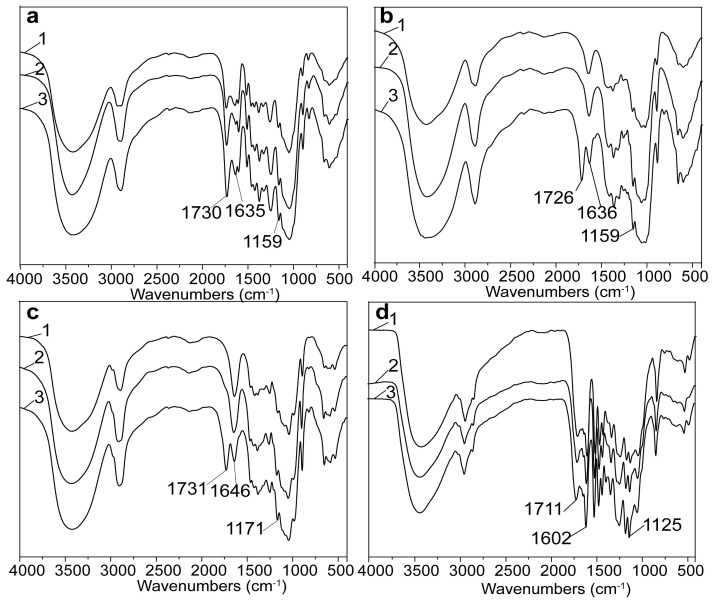
The Fourier translation infrared (FT-IR) spectra of unmodified (1), regenerated (2), and maleated (3) bagasse (**a**); cellulose (**b**); hemicelluloses (**c**); and, lignin (**d**) samples.

The structural changes of bagasse after maleation could be revealed by the FT-IR analysis, and the FT-IR spectra of unmodified, regenerated, and maleated samples are presented in Figure 1. Clearly, there was no difference between the FT-IR spectrum of regenerated bagasse and that of unmodified bagasse, indicating the unchanged skeleton of bagasse upon the dissolution and regeneration. The bands at 1730, 1635, and 1159 cm^−1^ are attributed to C=O stretching, C=C stretching, and C–O symmetric stretching, respectively [30,36]. After maleation, the enhanced intensity of these bands in the FT-IR spectrum of the maleated bagasse confirmed the occurrence of bagasse maleation [36,37]. However, due to the seriously overlapped absorptions of various components, the detailed structural changes of each component were difficult to be elucidated. Therefore, the maleated cellulose, hemicelluloses, and lignin were separately analyzed with FT-IR in order to elucidate the structural changes of each component, as shown in Figure 1b–d.

In Figure 1b, there were no obvious differences of the FT-IR spectra between the regenerated and unmodified cellulose, suggesting the non-derivatization of cellulose [38]. Comparatively, new absorptions at 1726, 1636, and 1159 cm^−1^ appeared in the maleated cellulose, indicating that maleoyl groups were attached onto cellulose [36]. The skeleton of the regenerated hemicelluloses was not changed, which was similar to the regenerated cellulose, and the appearance of the new absorptions at 1731, 1646, and 1171 cm^−1^ in the FT-IR spectrum of the maleated hemicelluloses (Figure 1c) also indicated the maleation of hemicelluloses [30,36]. Similarly, the unchanged skeleton of the regenerated lignin (Figure 1d) was observed, and the intensity of absorptions at 1711, 1602, and 1125 cm^−1^ in the FT-IR spectrum of the maleated lignin obviously increased, indicating the esterification of lignin [29]. These results showed that the skeleton of the three main components remained unchanged after dissolution in AmimCl, and that they were all involved in the maleation of bagasse in AmimCl.

### 3.3. Detailed Esterification of Reactive Sites during the Homogeneous Maleation

NMR, which is a versatile tool, is commonly used to qualitatively and quantitatively analyze chemical structures of samples. Especially, 2D NMR (^1^H-^1^H COSY, ^1^H-^13^C HSQC, and HMBC) could provide more detailed structural information and distinguish the overlapped peaks in 1D (^1^H and ^13^C) NMR. Since the signals of various components were seriously overlapped in the NMR spectra of bagasse samples [31,34], it was extremely difficult to obtain the detailed esterification information of the reactive sites in each component. Therefore, the maleated cellulose, hemicelluloses, and lignin samples were comparatively analyzed with the maleated bagasse by 1D and 2D NMR spectroscopy.

**Figure 2:**
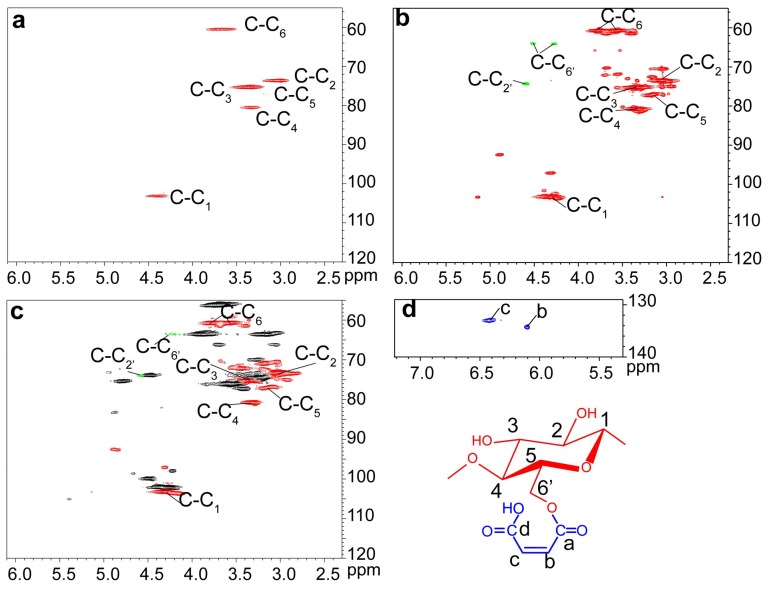
The ^1^H-^13^C heteronuclear single-quantum correlation (HSQC) nuclear magnetic resonance (NMR) spectra of unmodified cellulose (UC, (**a**)), maleated cellulose (MAC, (**b**,**d**)), and the cellulosic region of maleated bagasse (MAB, (**c**)).

The ^1^H-^13^C HSQC spectra of unmodified (UC, a) and maleated cellulose (MAC, b and d) as well as the cellulosic region of maleated bagasse (MAB, c) are presented in Figure 2. The cross-peaks for cellulose appeared in the range δ_C_/δ_H_ 120.00–55.00/6.10–2.30 ppm, and were well resolved based on their 1D (^1^H and ^13^C) NMR spectra (Figure S1) and the previous publications [37,39,40]. When compared to unmodified cellulose (Figure 2a), the correlation peaks from low-molecular cellulosic fractions obviously increased in the maleated cellulose (Figure 2b), indicating the degradation of cellulose macromolecules after homogeneous maleation [41]. In Figure 2b, the three signals from substituted C_2_ (C-C_2′_) and C_6_ (C-C_6′_) of anhydroglucose units (AGU) appeared at δ_C_/δ_H_ 73.61/4.54 (C_2′_/H_2′_), 64.11/4.52 (C_6′_/H_6′_), and 64.03/4.28 (C_6′_/H_6′_) ppm, respectively. In Figure 2d, the two correlation peaks at δ_C_/δ_H_ 133.27/6.40 and 134.22/6.10 ppm are assigned to C_c_/H_c_ and C_b_/H_b_ from the attached maleoyl group in the maleated cellulose. These suggested the predominant monoesterification of cellulose during the homogeneous maleation. Besides, the relative PS of hydroxyls at the different positions of AGU could be calculated by integrating the area of substituted and unsubstituted cross-peaks. In the maleated cellulose, the results showed that 50.9%, 49.1%, and 0 of maleoyl groups were attached to C_6_-OH, C_2_-OH, and C_3_-OH of AGU. In the maleated bagasse, the relative PS of C_6_-OH, C_2_-OH, and C_3_-OH on AGU were 52.6%, 47.3%, and 0, respectively. These indicated that the reactivity of hydroxyls of AGU during the homogeneous maleation followed the order of C_6-OH_ > C_2-OH_ > C_3-OH_, as reported in the previous study [42].

**Figure 3:**
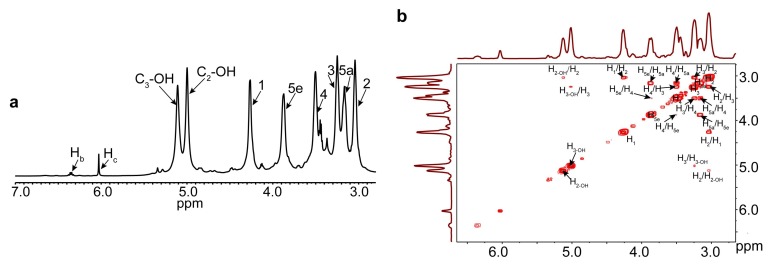

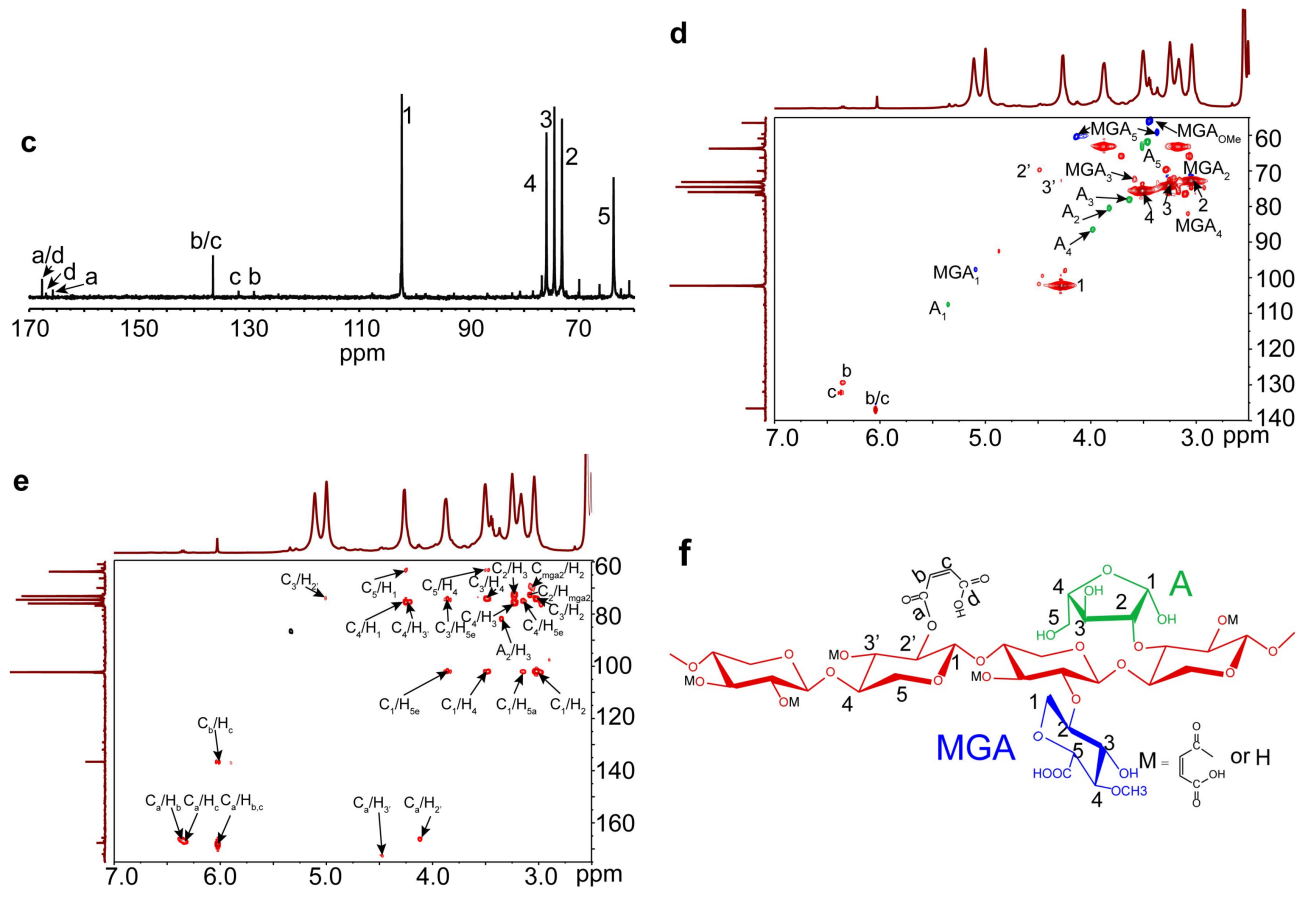
^1^H NMR (**a**); ^1^H-^1^H correlation spectroscopy (COSY) (**b**); ^13^C NMR (**c**); ^1^H-^13^C HSQC (**d**); and, heteronuclear multiple-bond correlation (HMBC) (**e**) spectra of the maleated hemicelluloses as well as the model (**f**) of the maleated hemicelluloses.

When considering the branched structure and the various sugar units of hemicelluloses, the ^1^H NMR (a), ^1^H-^1^H COSY (b), ^13^C NMR (c), ^1^H-^13^C HSQC (d), and HMBC (e) spectra of maleated hemicelluloses are presented in Figure 3 to reveal the detailed esterification information of reactive sites of hemicelluloses during the homogeneous maleation. The signals were assigned according to the previous literature [31]. In the ^1^H NMR spectra (Figure 3a), the proton peaks at 4.26, 3.04, 3.25, 3.50, 3.17, and 3.87 ppm are attributed to H_1_, H_2_, H_3_, H_4_, H_5a_, and H_5e_ of anhydroxylose units (AXU) in xylan, respectively. The proton peaks at 5.00 and 5.11 ppm relate to the protons from C_2-OH_ and C_3-OH_ of AXU. More importantly, two signals for protons of the maleoyl group appeared at 6.35 and 6.02 ppm, suggesting the attachment of maleoyl groups onto AXU. The ^1^H-^1^H COSY spectrum of the maleated hemicelluloses is presented in Figure 3b to confirm the assignment of proton peaks. The strong cross-peaks at δ_H_/δ_H_ 4.26/3.03, 3.03/3.25, 3.23/3.51, 3.53/3.14, 3.51/3.87, and 3.16/3.90 ppm are assigned to H_1_/H_2_, H_2_/H_3_, H_3_/H_4_, H_4_/H_5a_, H_4_/H_5e_, and H_5a_/H_5e_ of AXU, respectively, confirming the correct assignment of proton signals from xylan backbone. The ^13^C NMR spectrum of the maleated hemicelluloses is shown in Figure 3c. Five primary carbon signals at 102.25, 73.12, 74.51, 75.94, and 63.74 ppm are associated with C_1_, C_2_, C_3_, C_4_, and C_5_ of AXU, respectively. More importantly, the peaks at 165.62, 129.13, 131.90, and 166.92 ppm for C_a_, C_b_, C_c_, and C_d_ of maleoyl carbons, respectively, in the monoesterified hemicelluloses were well resolved, and two signals at 167.67 and 136.59 ppm for C_a/d_ and C_b/c_ of maleoyl carbons in the diesterified hemicelluloses were also clearly observed. These results were similar to the maleated xylan that was prepared in the 1-butyl-3-methylimidazolium chloride [21]. Figure 3d illustrates the ^1^H-^13^C HSQC spectrum of maleated hemicelluloses. The strong anomeric cross-peaks at δ_C_/δ_H_ 102.31/4.27, 97.82/5.08, and 107.62/5.35 ppm are attributed to C_1_/H_1_ of xylan, 4-*O*-methyl-d-glutaric acid (MGA), and arabinose (A), respectively. These confirmed that the isolated hemicelluloses were mainly composed of arabino-4-*O*-methylglucuronicxylan, as consistent with the reported literature [43]. The cross-peaks at δ_C_/δ_H_ 129.21/6.33 and 131.73/6.37 ppm are originated from C_b_/H_b_ and C_c_/H_c_ in the monoesterified hemicelluloses, and the cross-peak at δ_C_/δ_H_ 136.60/6.03 ppm relates to C_b,c_/H_b,c_ in the diesterified hemicelluloses, corresponding to the assignment of peaks in the ^1^H and ^13^C NMR spectra. The ratio of monoester to diester in the maleated hemicelluloses was 1:1.5 by integrating the resonances that were assigned to C_b_/H_b_ and C_b,c_/H_b,c_. More importantly, two correlations at δ_C_/δ_H_ 70.10/4.48 and 73.13/4.28 ppm are for C_2′_/H_2′_ and C_3′_/H_3′_ from the substituted C_2_ (2′ in Figure 3d) and C_3_ (3′ in Figure 3d) of AXU. Similar to cellulose samples, the relative PS of reactive sites of AXU could also be calculated according to the integral area of the substituted and unsubstituted correlation peaks. The results showed that 56.9% and 43.1% of maleoyl groups were attached to C_2_-OH and C_3_-OH of AXU, respectively, suggesting the higher reactivity of hydroxyls at C_2_ of AXU during the homogeneous maleation. In addition, the correlation peaks at δ_C_/δ_H_ 92.50/4.86 (C_α1_/H_α1_), 98.03/4.24 (C*_β_*_1_/H*_β_*_1_), and 102.31/4.27 (C_NR1_/H_NR1_) ppm from *α*-, *β*- xylan reducing end and non-reducing end units were clearly identified. When compared with unmodified hemicelluloses (Figure S2), these noticeably increased correlations in the ^1^H-^13^C HSQC spectrum of the maleated hemicelluloses indicated the degradation of hemicelluloses during the homogenous maleation. According to our previous study [31], the contents of the hemicellulose main-chains and side-chains could be compared by integrating resonances assigned to xylose and arabinose. The ratio of xylose to arabinose was 29:1 in the unmodified hemicelluloses, and largely increased to 97:1 in the maleated hemicelluloses, confirming the degradation of hemicelluloses during the homogeneous maleation. This degradation was possibly due to the released maleic acid from maleic anhydride upon esterification in AmimCl [34]. HMBC could be used to further prove the maleation of xylan and the correct attribution of the primary signals of the maleated hemicelluloses [44]. The HMBC spectrum of the maleated hemicelluloses is illustrated in Figure 3e. Expectedly, the correlations between carbonyl carbon (C=O) in the maleoyl group and the protons at the substituted C_3_ and C_2_ (3′ and 2′ protons) of AXU are present at δ_C_/δ_H_ 165.63/4.12 and 171.88/4.49 ppm, respectively. These signals indicated the chemical bonding between the maleoyl group and xylan, thus confirming the attachment of maleoyl group onto xylan. In addition, the cross-peaks for the correlations between carbonyl carbon and protons of the maleoyl group appeared at δ_C_/δ_H_ 166.37/6.37 (C_a_/H_b_), 166.84/6.34 (C_a_/H_c_), and 167.67/6.03 (C_a_/H_b,c_) ppm, confirming the correct assignment of cross-peaks in the ^1^H-^13^C HSQC spectrum. In addition, two cross-peaks at δ_C_/δ_H_ 70.49/3.24 (C_MGA2_/H_2_) and 72.88/3.10 (C_2_/H_MGA2_) suggested that MGA were attached to C_2_ of AXU. The cross peaks at δ_C_/δ_H_ 82.25/3.36 ppm for C_A2_/H_3_ indicated that the arabinose units were linked to C_3_ of AXU. Two correlations at δ_C_/δ_H_ 75.95/4.26 (C_4_/H_1_) and 102.15/3.50 (C_1_/H_4_) belong to the linked xylose units by *β*-1,4 linkages. Based on these results, the model of the maleated hemicelluloses is shown in Figure 3f. In the ^1^H-^13^C HSQC spectrum of the hemicellulosic region of the maleated bagasse (Figure S3), the primary correlation peaks from xylan were well resolved according to the ^1^H-^13^C HSQC spectra of the unmodified and maleated hemicelluloses. By integration, the relative PS at C_2_ and C_3_ of AXU were 49.5% and 50.5%, suggesting the similar reactivity of AXU hydroxyls of bagasse during the homogeneous esterification. This was different from the isolated hemicelluloses, which was probably due to the higher reactivity of hydroxyls at C_2_ of AXU in forming quinone methide intermediate during the biosynthesis of lignin carbohydrate complex in the whole plant cell walls [45].

Due to the existence of various hydroxyls, it is necessary to analyze the reactive sites of lignin during the homogeneous maleation with ^31^P NMR spectroscopy. The contents of different hydroxyls in lignin samples were calculated from the ^31^P NMR spectra (Figure S4), as listed in Table 2. After maleation, the content of aliphatic hydroxyl obviously decreased from 3.96 (UL) to 3.34 mmol/g (MAL), and the content of the total phenolic hydroxyl decreased from 1.10 (UL) to 1.01 mmol/g (MAL). These decreases suggested that both aliphatic and phenolic hydroxyls of lignin were maleated during the homogeneous esterification. The similar results were also reported in the esterification of pine lignin with butyric anhydride [46]. In the maleated lignin, the decreased percentage of aliphatic hydroxyls (15.7%) was higher than that of phenolic ones (8.2%), indicating that aliphatic hydroxyls were more reactive than phenolic hydroxyls. For different phenolic hydroxyls, the decreased percentage of *p*-hydroxyphenyl OH (11.8%) was a little higher than that of guaiacyl OH (11.5%), suggesting the slightly higher reactivity of *p*-hydroxyphenyl OH. However, a slight increase in the content of syringyl OH was observed. According to the previous literature [47], the *β*-*O*-4′ linkages are mainly formed by syringyl OH. These indicated that the *β*-*O*-4′ linkages were broken and the new syringyl OH was released during the homogeneous meleation. Besides, an increase of carboxyl content from 0.11 (UL) to 0.34 mmol/g (MAL) further confirmed the esterification of lignin. However, in the maleated lignin, the decreased hydroxyl content was much higher than the increased carboxyl content, which was probably due to the predominant diesterification of lignin during the homogeneous maleation.

**Figure 4:**
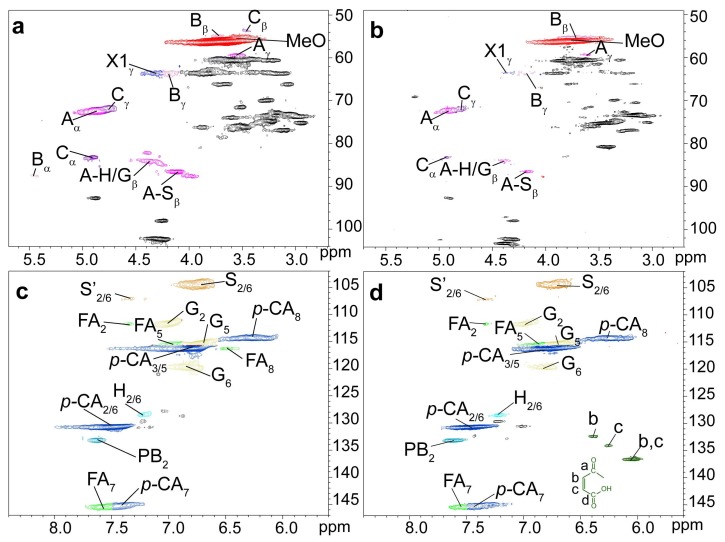
The ^1^H-^13^C HSQC NMR spectra of unmodified (UL, (**a**,**c**)) and maleated lignin (MAL, (**b**,**d**)).

**Figure 5:**
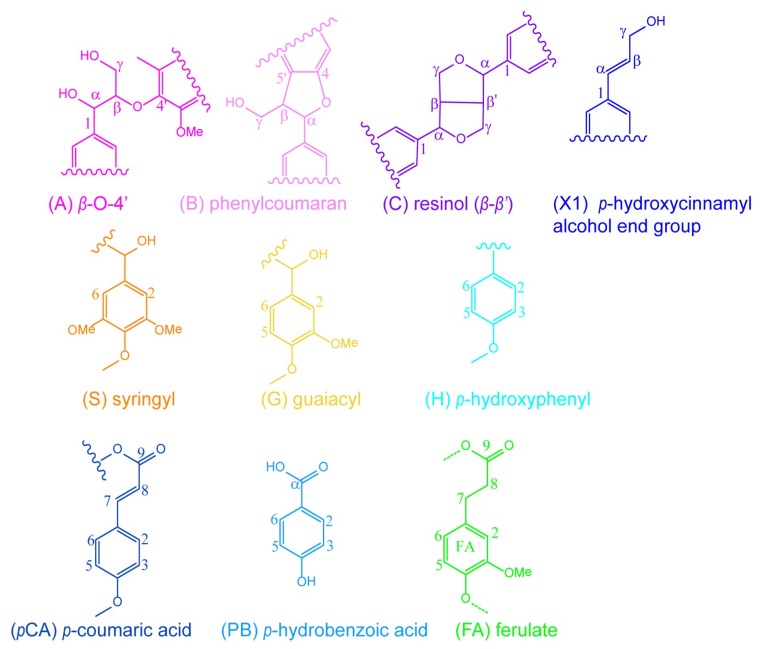
The substructures of lignin isolated from bagasse.

The ^1^H-^13^C HSQC spectra of unmodified (UL, a and c) and maleated lignin (MAL, b and d) are presented in Figure 4, and the main substructures of the isolated lignin are presented in Figure 5. The ^1^H-^13^C HSQC spectra of lignin samples were divided into two regions: side-chains region (δ_C_/δ_H_ 108.00-48.00/5.70-2.70 ppm) and aromatic region (δ_C_/δ_H_ 147.00-102.00/8.40-5.60 ppm). The primary cross-peaks of lignin were well assigned, according to their 1D (^1^H and ^13^C) NMR spectra (Figure S5) and the previous literatures [48,49]. In the aromatic region, the cross-peaks from S, G, H, ferulate (FA), and *p*-coumaric acid (*p*CA) could be well recognized. The main lignin inter-unit linkages, including *β*-aryl ethers (*β-O*-4′, A), phenylcoumaran (*β*-5′, B), and resinol (*β*-*β*′, C) could be well distinguished in lignin side-chains region. The cross-peak for *p*-hydroxycinnamyl alcohol end group (X1) was also well resolved. The chemical shifts of these identified signals are shown in Table S1. More importantly, the correlation peaks for C_b_/H_b_ and C_c_/H_c_ of the maleoyl groups in the monoesterified lignin appeared at δ_C_/δ_H_ 132.43/6.39 and 134.05/6.26 ppm, respectively, and the cross-peak for C_b,c_/H_b,c_ of maleoyl group in the diesterified lignin was present at δ_C_/δ_H_ 136.63/6.03 ppm. By integration, the ratio of monoester to diester in the maleated lignin was 1:5.9, suggesting the predominant diesterification of lignin during the homogeneous maleation.

According to the previous literature [50], the semi-quantification method based on ^1^H-^13^C HSQC NMR was adopted to quantify the content of lignin substructures. The detailed information of lignin substructures was calculated from ^1^H-^13^C HSQC spectra, as shown in Table 3. After maleation, the relative molar quantity of *β*-*O*-4′ aryl ether obviously decreased from 45.6/100Ar (UL) to 37.7/100Ar (MAL), suggesting the cleavage of *β*-*O*-4′ aryl ether linkages during the homogeneous maleation [51]. In the meantime, the decreases in the contents of resinol and phenylcoumaran linkages were also observed. These results indicated the degradation of lignin side-chains during the homogeneous maleation in AmimCl. In the aromatic region, the relative content of G-units decreased from 43.7% (UL) to 40.6% (MAL), and the relative content of H-units also reduced from 7.5% (UL) to 6.6% (MAL). These results indicated the easy degradation of G- and H-units in the homogeneous maleation [52], which probably resulted from the maleic acid that was released from maleic anhydride in AmimCl [53]. The relative molar quantity of *p*CA increased from 131.3/100Ar (UL) to 141.6/100Ar (MAL), and the relative molar quantity of FA also increased from 8.3/100Ar (UL) to 11.9/100Ar (MAL). These were possibly due to the degradation of lignin aromatic units, which led to the decreased intensity of C_2,6_/H_2,6_ correlations from G- and H-units during the homogeneous maleation.

**Figure 6:**
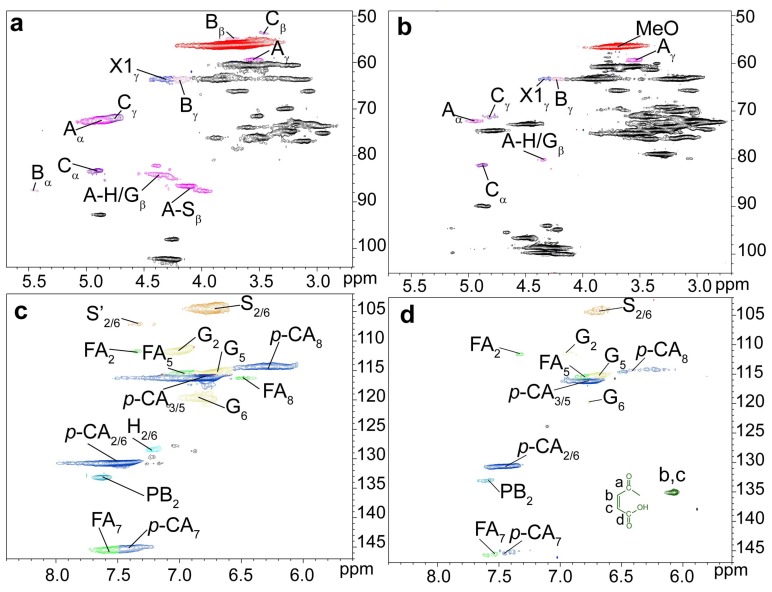
The ^1^H-^13^C HSQC NMR spectra of unmodified lignin (UL, (**a**,**c**)) and lignin region of maleated bagasse (MAB, (**b**,**d**)).

The ^1^H-^13^C HSQC spectra of unmodified lignin (UL, a and c) and the lignin region of maleated bagasse (MAB, b and d) are depicted in Figure 6, and the main substructures of lignin are presented in Figure 5. The cross-peak at δ_C_/δ_H_ 135.03/6.07 ppm for C_b,c_/H_b,c_ of maleoyl groups in the maleated bagasse further confirmed the maleation between bagasse and maleic anhydride, as consistent with the FT-IR analysis. The relative molar quantities of *β*-*O*-4′ aryl ether and phenylcoumaran linkages in the maleated bagasse both decreased by semi-quantitative ^1^H-^13^C HSQC analysis. This suggested the cleavage of *β*-*O*-4′ aryl ether and phenylcoumaran linkages during the homogeneous maleation of bagasse. The relative contents of G- and H-units also decreased in the maleated bagasse, thus indicating the degradation of lignin aromatic units (G- and H-units) in the homogeneous maleation of bagasse. When compared with those in the unmodified lignin, the relative molar quantities of *p*CA and FA increased in the maleated bagasse, which was consistent with the maleated lignin.

### 3.4. XPS Analysis of Bagasse Samples

**Figure 7:**
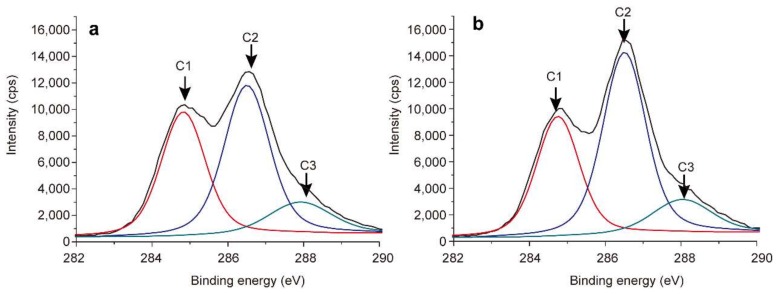
C1s spectra of unmodified (**a**) and maleated (**b**) bagasse.

Figure 7 presents the deconvoluted C1s signals of unmodified (a) and maleated (b) bagasse samples. The C1s peak was deconvoluted into three subpeaks: C1 at 284.8 eV is assigned to CC, CH, and C=C; C2 at 286.5 eV corresponds to C–O and O–C–O; C3 at 287.9eV is related with C=O [54,55]. The XPS results of the resolved C1s peaks of samples are listed in Table 4. After maleation, the contents of C1 (C–C, C–H, or C=C) and C3 (C=O) increased from 34.6% and 13.5% (UB) to 38.0% and 14.0% (MAB), respectively. This was resulting from the introduced maleoyl groups and the formation of new ester bonds. These results further confirmed the occurrence of esterification between bagasse and maleic anhydride, which was consistent with those from FT-IR and NMR analyses.

## 4. Conclusions

The three main components (cellulose, hemicelluloses, and lignin) of bagasse were all involved in the homogeneous esterification, and the reactivity followed the order of lignin > cellulose > hemicelluloses. NMR analysis indicated that the predominant monoesterification of cellulose, the diesterification of hemicelluloses and lignin, and the degradation of the three main components simultaneously occurred during the maleation. ^1^H-^13^C HSQC analysis indicated that 50.9%, 49.1%, and 0 of maleoyl groups were attached to C_6_-OH, C_2_-OH, and C_3_-OH of AGU of the maleated cellulose. In the maleated hemicelluloses, the ratio of monoester to diester was 1:1.5, and the relative PS of hydroxyls at C_2_ and C_3_ of AXU were 56.9% and 43.1%. The ratio of monoester to diester of the maleated lignin was 1:5.9, and the aliphatic hydroxyls were more reactive than the phenolic hydroxyls. Besides, the reactivity of the phenolic hydroxyls of lignin followed the order of *p*-hydroxyphenyl OH > guaiacyl OH > syringyl OH. These structural changes of bagasse during the homogeneous maleation are beneficial to effectively tune the PS, homogeneity, and the compatibility of composites based on the maleated bagasse.

## Figures and Tables

**Table 1:** The hydroxyl contents and the percentages of substitution of the maleated bagasse, cellulose, hemicelluloses, and lignin.

Sample *^a^*	Reaction Conditions			n’_OH_ *^b^* (mmol/g)	n_OH_ *^c^* (mmol/g)	PS *^d^* (%)
	MA Dosage (g/g)	Temperature (°C)	Reaction Time (min)			
MAB	5:1	90	90	1.79	14.30	12.52
MAC	5:1	90	90	2.50	18.52	13.50
MAH	5:1	90	90	1.65	15.15	10.89
MAL	5:1	90	90	0.71	5.06	14.03

*^a^* The maleated bagasse, cellulose, hemicelluloses, and lignin, respectively; *^b^* The substituted hydroxyl content; *^c^* The theoretical hydroxyl content; *^d^* The percentage of substitution.

**Table 2:** The hydroxyl content of unmodified (UL) and maleated lignin (MAL).

Samples	UL	MAL
Aliphatic OH (mmol/g)	3.96	3.34
Total phenolic OH (mmol/g)	1.10	1.01
Syringyl (S) OH (mmol/g)	0.08	0.11
Guaiacyl (G) OH (mmol/g)	0.26	0.23
*p*-Hydroxyphenyl (H) OH (mmol/g)	0.76	0.67
Carboxyl (mmol/g)	0.11	0.34
S/G ratio	0.31	0.48

**Table 3:** Quantitative composition information of lignin from the ^1^H-^13^C HSQC NMR spectra of unmodified lignin (UL), maleated lignin (MAL), and maleated bagasse (MAB).

Samples	UL	MAL	MAB
**Results expressed per 100 Ar**			
Aryl ether (A)	45.6	37.7	27.7
Phenylcoumaran (B)	3.5	0.9	2.5
Resinol (C)	9.7	8.5	12.6
**S + G + H = 100%**			
Guaiacyl (G)	43.7%	40.6%	32.7%
Syringyl (S)	48.7%	52.8%	62.9%
*p*-Hydroxyphenyl (H)	7.5%	6.6%	4.4%
**Results expressed per 100 Ar**			
*p*-Coumarate acid (*p*CA)	131.3	141.6	141.5
Ferulate (FA)	8.3	11.9	11.4

**Table 4:** XPS results from the resolved C1s peaks of unmodified (UB) and maleated (MAB) bagasse.

Samples	C1% (C–C, C–H, C=C)	C2% (C–O, O–C–O)	C3% (C=O)
UB	34.6	51.9	13.5
MAB	38.0	48.0	14.0

## Supplementary Materials

The following are available online at http://www.mdpi.com/2073-4360/10/4/433/s1. The detailed parameters for NMR and XPS analyses were listed in the section of instrumental parameters. Figure S1: The ^1^H and ^13^C NMR spectra of unmodified (a,c) and maleated (b,d) cellulose, Figure S2: The ^1^H-^13^C HSQC spectrum of unmodified hemicelluloses, Figure S3: The hemicellulosic region in the ^1^H-^13^C HSQC spectrum of the maleated bagasse, Figure S4: The ^31^P NMR spectra of unmodified (a) and maleated lignin (b), Figure S5: The ^1^H and ^13^C NMR spectra of unmodified (a,c) and maleated lignin (b,d), Table S1: Assignment of ^13^C/^1^H cross-peaks in the ^1^H-^13^C HSQC spectra of lignin samples.
