# Supplementary material for: Structural Changes of Bagasse during the Homogeneous Esterification with Maleic Anhydride in Ionic Liquid 1-Allyl-3-methylimidazolium Chloride
Source: Polymers (Basel). 2018 Apr 13;10(4):433. doi: 10.3390/polym10040433 (PMC6415262; doi:10.3390/polym10040433)
Supplement: Supplementary file 1 [file polymers-10-00433-s001.pdf]

Structural Changes of Bagasse during the Homogeneous Esterification with Maleic Anhydride in Ionic Liquid 1-Allyl-3-methylimidazolium Chloride

Huihui Wang [1], Wei Chen [1], Xueqin Zhang [1], Yi Wei [1], Aiping Zhang [2], Shijie Liu [1,3,*], Xiaoying Wang [1] and Chuanfu Liu [1,*]

1 State Key Laboratory of Pulp and Paper Engineering, South China University of Technology, Guangzhou 510640, P. R. China; wang.huihui@mail.scut.edu.cn (H.W.); geogeo_chen@163.com (W.C.); xueqin0228@gmail.com (X.Z); fewvergil@mail.scut.edu.cn (Y.W.); sliu@esf.edu (S.L.); xyw@scut.edu.cn (X.W.); chfliu@scut.edu.cn (C.L.);

2 College of Forestry and Landscape Architecture, South China Agricultural University, Guangzhou, 510642, China; aiping@scau.edu.cn

3 Department of Paper and Bioprocess Engineering, College of Environmental Science and Forestry, State University of New York, Syracuse, NY 13210, USA; sliu@esf.edu (S.L.)

* Correspondence: sliu@esf.edu (S.L.); chfliu@scut.edu.cn (C.L.); Tel.: +86-20-87113912 (C.L.)

Instrumental Parameters

The detailed collecting and processing parameters for ^{1}H NMR analysis were listed as follows: number of scans, 16; receiver gain, 31; acquisition time, 2.7263 s; relaxation delay, 1.0 s; pulse width, 11.0 s; spectrometer frequency, 600.17 MHz; and spectral width, 12019.2 Hz. The detailed collecting and processing parameters for ^{1}H-^{1}H COSY NMR analysis were listed as follows: number of scans, 16; receiver gain, 14; acquisition time, 2.0090 s; relaxation delay, 12.40 s; pulse width, 0.1188 s; spectrometer frequency, 600.17/600.17 MHz; and spectral width, 8620.7/8620.7 Hz. The detailed collecting and processing parameters for ^{13}C NMR analysis were listed as follows: number of scans, 1452; receiver gain, 187; acquisition time, 0.9088 s; relaxation delay, 2.0 s; pulse width, 12.0 s; spectrometer frequency, 150.91 MHz; and spectral width, 36057.7 Hz. The detailed collecting and processing parameters for ^{1}H-^{13}C HSQC analysis were listed as follows: number of scans, 32; receiver gain, 187; relaxation delay, 1.5 s; pulse width, 11.0 s; acquisition time, 0.1420 s; spectrometer frequency, 600.17/150.91MHz; and spectral width 7211.5/24875.6 Hz. The detailed collecting and processing parameters for ^{1}H-^{13}C HMBC NMR analysis were listed as follows: number of scans, 134; receiver gain, 187; acquisition time, 1.5123 s; relaxation delay, 12.40 s; pulse width, 0.1188 s; spectrometer frequency, 600.17/150.91 MHz; and spectral width, 8620.7/33557.0 Hz. The detailed XPS parameters were listed as follows: total acquisition time, 68.0 s; number of scans, 1; pass energy, 100.0 eV; energy step size, 1.000 eV; number of energy steps, 1361; and spot size, 650 μm.

Table S1. Assignment of $^{13}C/^{1}H$ cross-peaks in the ^{1}H-^{13}C HSQC spectra of lignin samples

Lable	δ_C/δ_H(ppm)[a]	δ_C/δ_H(ppm)[b]	Assignments
A_α	72.43/4.84	72.35/4.86	C_α/H_α in *β*-*O*-4′ substructures
A_γ	60.35/3.48	63.57/4.31	C_γ/H_γ in*β*-*O*-4′ substructures
A-G/H_β	84.16/4.35	84.03/4.34	C_β/H_β in*β*-*O*-4′ linked to G/H
A-S_β	86.69/4.09	86.56/4.10	C_β/H_β in*β*-*O*-4′ linked to S
B_α	87.67/5.43	--	C_α/H_α in phenylcoumaran (B)
B_β	--	55.36/3.70	C_β/H_β in phenylcoumaran (B)
B_γ	63.50/4.21	63.57/4.07	C_γ/H_γ in phenylcoumaran (B)
C_α	83.59/4.92	83.05/4.88	C_α/H_α in *β*-*β*′ (resinol) (C)
C_β	53.73/3.45	--	C_β/H_β in *β*-*β*′ (resinol) (C)
C_γ	71.86/4.74	72.15/4.84	C_γ/H_γ in *β*-*β*′ (resinol) (C)
$X1_\gamma$	63.70/4.30	63.57/4.31	C_γ/H_γ in cinnamyl alcohol end-group (X1)
$S_{2,6}$	104.68/6.72	104.61/6.68	$C_{2,6}/H_{2,6}$ in syringyl units (S)
$S'_{2,6}$	107.15/7.34	106.94/7.31	$C_{2,6}/H_{2,6}$ in oxidized S units (S′)
G_2	111.69/7.00	111.78/6.99	C_2/H_2 in guaiacyl units (G)
G'_2	111.84/7.35	111.62/7.32	C_2/H_2 in oxidized G units (G′)
G_5	115.33/6.71	115.15/6.68	C_5/H_5 in guaiacyl units (G)
G_6	119.75/6.80	119.68/6.81	C_6/H_6 in guaiacyl units (G)
$H_{2,6}$	118.66/7.20	118.59/7.20	$C_{2,6}/H_{2,6}$ in p-hydroxyphenyl (H)
$PCA_{2,6}$	130.98/7.47	130.65/7.46	$C_{2,6}/H_{2,6}$ in p-hydrobenzonic acid (PCA)
$PCA_{3,5}$	116.38/6.77	116.16/6.76	$C_{3,5}/H_{3,5}$ in p-coumaric acid (PCA)
PCA_7	145.32/7.41	145.41/7.41	C_7/H_7 in p-coumaric acid (PCA)
PCA_8	114.48/6.27	114.41/6.25	C_8/H_8 in p-coumaric acid (PCA)
$PB_{2,6}$	133.19/7.62	133.13/7.60	$C_{2,6}/H_{2,6}$ in p-hydrobenzonic acid (PB)
FA_2	111.84/7.34	111.62/7.33	$C_{2,6}/H_{2,6}$ in ferulate (FA)
FA_5	115.64/6.93	115.58/6.88	$C_{2,6}/H_{2,6}$ in ferulate (FA)
FA_7	145.32/7.40	145.41/7.41	$C_{2,6}/H_{2,6}$ in ferulate (FA)

[a]The chemical shifts of unmodified lignin. [b]The chemical shifts of maleated lignin.

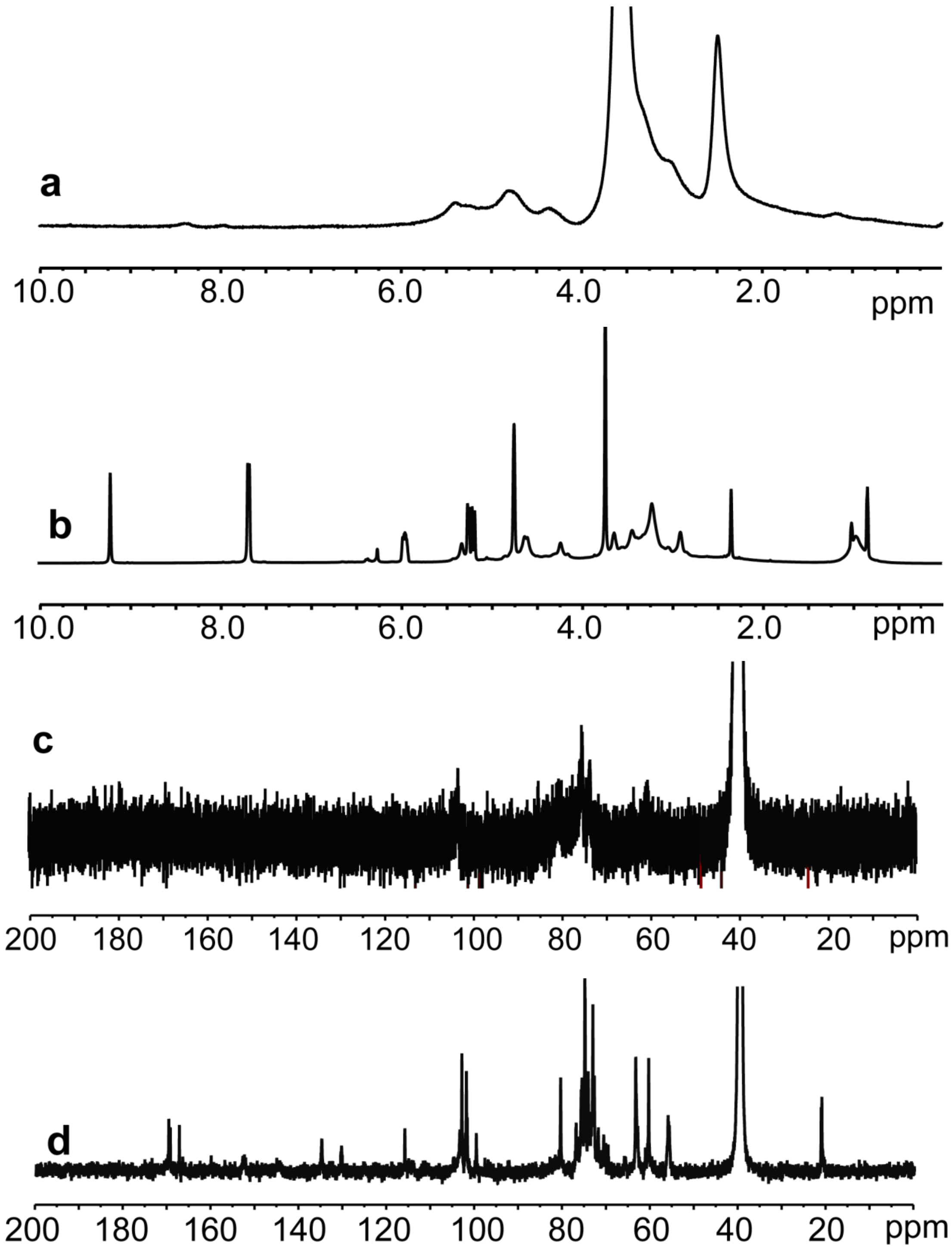

Figure S1. The ^{1}H and ^{13}C NMR spectra of unmodified (a,c) and maleated (b,d) cellulose.

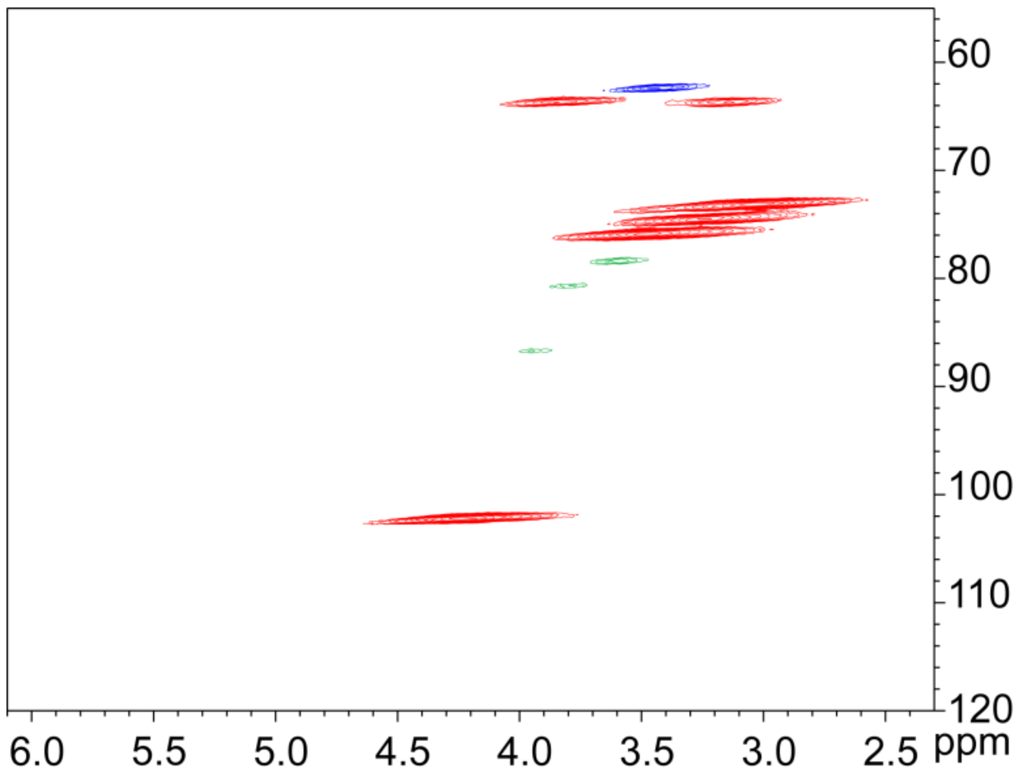

Figure S2. The ^{1}H-^{13}C HSQC spectrum of unmodified hemicelluloses.

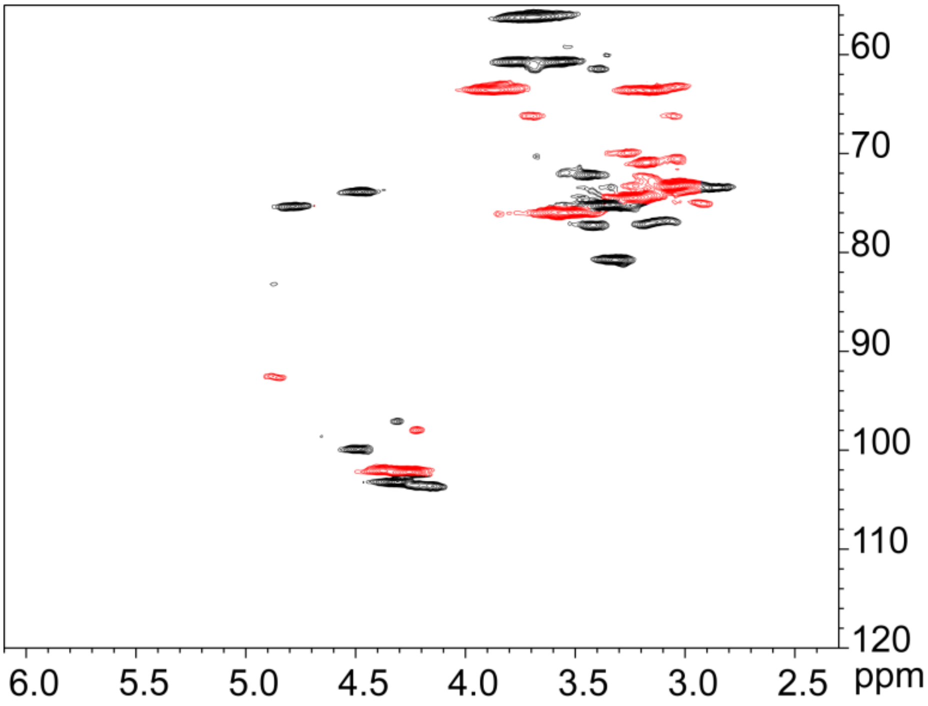

Figure S3. The hemicellulosic region in the ^{1}H-^{13}C HSQC spectrum of the maleated bagasse.

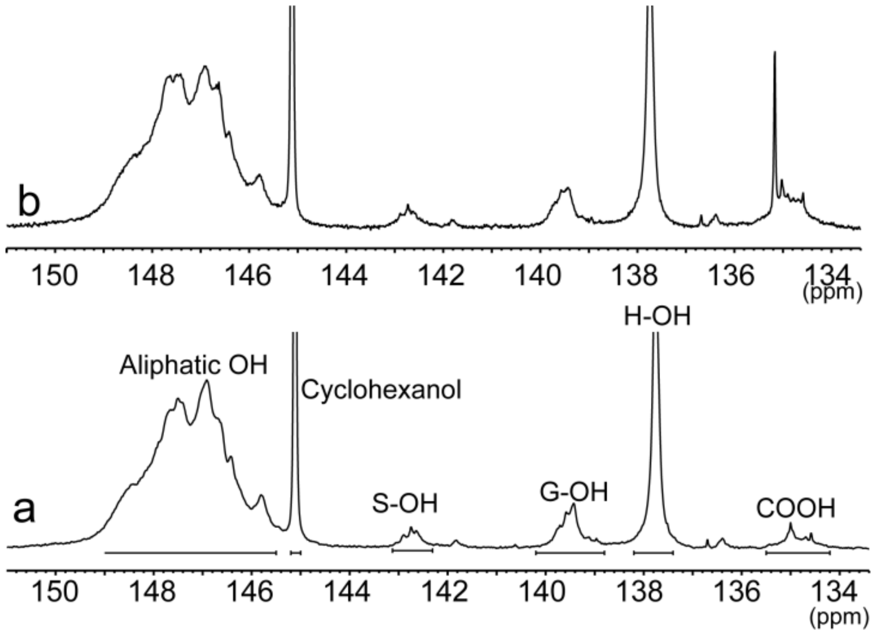

Figure S4. The ^{31}P NMR spectra of unmodified (a) and maleated lignin (b).

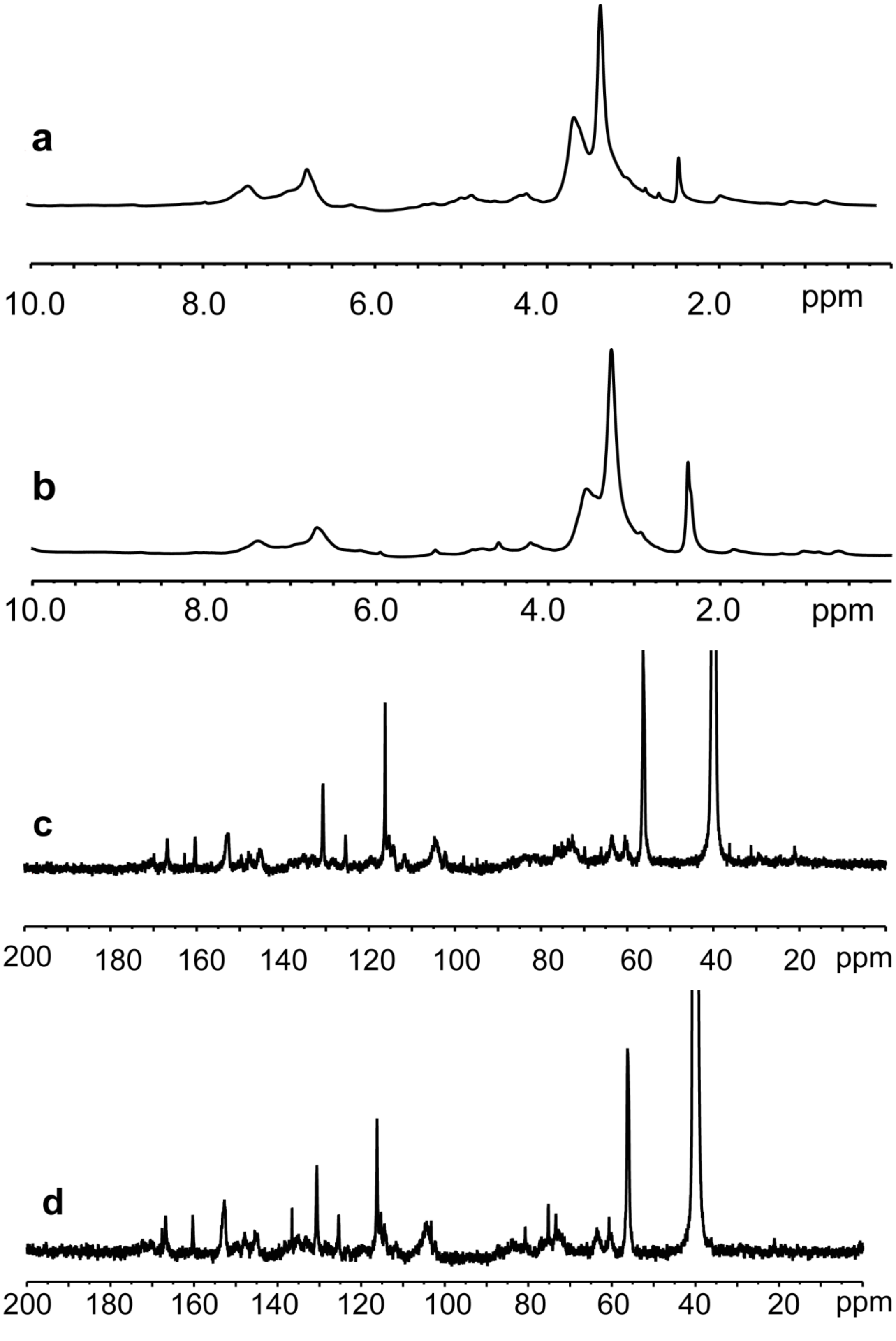

Figure S5. The ^{1}H and ^{13}C NMR spectra of unmodified (a,c) and maleated lignin (b,d).
